# An HDAC9-associated immune-related signature predicts bladder cancer prognosis

**DOI:** 10.1371/journal.pone.0264527

**Published:** 2022-03-03

**Authors:** Yang Fu, Shanshan Sun, Jianbin Bi, Chuize Kong, Du Shi

**Affiliations:** 1 Department of Urology, The First Hospital of China Medical University, Shenyang, PR China; 2 Department of Pharmacy, People’s Hospital Affiliated of China Medical University, Shenyang, PR China; Seoul National University College of Pharmacy, REPUBLIC OF KOREA

## Abstract

**Background:**

The close relationship between histone deacetylase 9 (HDAC9) and immunity has attracted attention. We constructed an immune signature for HDAC9, a vital epigenetic modification, to predict the survival status and treatment benefits in bladder cancer (BC).

**Methods:**

An exhaustive analysis of HDAC9 and immunology via the tumor and immune system interaction database (TISIDB) was performed, and an immune prognostic risk signature was developed based on genes enriched in the top five immune-related pathways under high HDAC9 status. Comprehensive analysis of survival curves and Cox regression were used to estimate the effectiveness of the risk signature. The relationship between immunological characteristics and the risk score was evaluated, and the mechanisms were also explored.

**Results:**

In the TISIDB, HDAC9 was closely related to various immunological characteristics. The risk signature was obtained based on genes related to prognosis enriched in the top five immune-related pathways under high HDAC9 status. The survival rate of the high-risk BC patients was poor. The risk score was closely related to multiple immunological characteristics, drug sensitivity, immunotherapy benefits and biofunctions.

**Conclusion:**

An immune-related prognostic signature established for HDAC9 expression status could independently predict the prognosis of BC patients. The use of this signature could help clinicians make personalized treatment decisions.

## Background

As the most common urinary tumor in the whole world, the morbidity of bladder cancer (BC) is increasing each year in China [1]. Once BC progresses to the advanced stage of metastasis, conventional treatments are no longer effective [2, 3]. Furthermore, the clinical outcome of BC in the past few years has been poor because of high recurrence and drug resistance rates [4]. Thus, identifying new markers for prognosis and treatment for BC is necessary.

Histone deacetylation is considered a vital epigenetic modification in vivo. Histone deacetylases (HDACs) restrain related gene expression by catalyzing the deacetylation of histones or nonhistone proteins [5–7]. Histone deacetylase 9 (HDAC9) is a IIa HDAC subtype that has shown dual roles in tumors in multiple studies. In pancreatic ductal adenocarcinoma, high HDAC9 expression was significantly related to poor prognosis [8], and overexpression of HDAC9 promoted progression of the malignant phenotype of oral squamous cell carcinoma [9]. Additionally, elevated HDAC9 expression activated angiogenesis and invasion in triple-negative breast cancer [10]. However, HDAC9 also exhibited inhibitory effects in tumors. HDAC9 stimulated the expression of the ATDC target gene P53 by inhibiting ATDC expression, leading to inhibition of tumorigenesis [11], and it suppressed the proliferation of gastric cancer cells and enhanced sensitivity to cisplatin [12]. Our previous study suggested that low HDAC9 expression might facilitate clear cell renal cell carcinoma (ccRCC) cell growth, and we further found that HDAC9 was closely related to immunity and increased the infiltration levels of a variety of immune cells [13]. Other studies have revealed that HDAC9 deficiency significantly suppresses the immune response and inflammatory response [14–16]. Currently, immunotherapy has great potential in BC, and several immune-related drugs are currently in development [17]. Therefore, we sought to explore the pathogenesis of HDAC9 in BC, especially the relationship between HDAC9 and immunological characteristics.

In this study, a comprehensive analysis of HDAC9 was carried out to explore the connection between HDAC9 and the immune phenotype in BC. Importantly, we constructed an immune signature according to the expression status of HDAC9, a vital epigenetic modification, to predict the survival status and treatment benefits in BC.

## Methods

### Data acquisition from multiple public databases

The mRNA and clinically related factor data were obtained from The Cancer Genome Atlas (TCGA) database (https://portal.gdc.cancer.gov/), GSE32548 from the Gene Expression Omnibus (GEO) database (https://www.ncbi.nlm.nih.gov/geo/) and the IMvigor210 trial [18]. TCGA data comprised the training cohort, GEO data were used as the validation cohort, and IMvigor210 trial data were used to evaluate the effect of immunotherapy. Immunohistochemical images of HDAC9 in BC samples and normal samples were retrieved from the Human Protein Atlas (HPA) database (https://www.proteinatlas.org/). The HDAC9 antibody product number used on the HPA website was HPA028926 (Atlas Inc., Stockholm, Sweden). Then, the connections between HDAC9 and the immune phenotypes were studied via the tumor and immune system interaction database (TISIDB) (http://cis.hku.hk/TISIDB/index.php) [19].

### Gene set enrichment analysis (GSEA)

BC samples in the TCGA database were split into two groups: a high HDAC9 expression group and a low HDAC9 group. Immune-related biological pathways were distinguished via GSEA through a “c5.all.v7.4.symbols.gmt” gene set downloaded from the Molecular Signatures Database (MSigDB). A nominal P value (NOM P value) < 0.05 and a false discovery rate Q value (FDR Q value) < 0.25 were considered to indicate significant enrichment, and genes in the top five enriched immune-related biological pathways were extracted.

### Risk score calculation

Genes related to survival were distinguished via univariable Cox regression. The P value was corrected for multiple comparisons by the FDR method, and a P adjusted value (P. adj) < 0.05 was considered statistically significant. Then, through least absolute shrinkage and selection operator (LASSO) Cox regression (the glmnet package of R), a risk score was calculated. The BC samples were split into two risk groups (low-risk vs. high-risk). A receiver operating characteristic (ROC) curve was utilized to analyze the risk signature accuracy for predicting prognosis via the R timeROC package, and the overall survival (OS) of the two groups was compared via a survival curve using the R survival package. To assess the ability of the risk score to independently predict prognosis, Cox regressions were then performed. The performance of prognosis prediction was also assessed through a nomogram and calibrations via the rms R package. To compare the predictive ability of our risk signature to other published signatures, we included risk signatures based only on prognostic-related genes [20–23] and those based on other risk factors [24–28] in the comparison of the AUCs of the ROC curves via the timeROC R package.

### Coexpression analysis

Coexpression analysis was performed to further understand connections between HDAC9 and the risk score genes via the psych package of R. The relevant results were visualized using the ggplot2 R package. The P value was corrected for multiple comparisons by the FDR method, and P. adj < 0.05 was considered statistically significant.

### The infiltration of immune cells and the risk score

To appraise the immune infiltration levels between two different risk groups, cell-type identification by estimating relative subsets of RNA transcripts (CIBERSORT) was carried out. In a previous study, CIBERSORT could calculate the relative abundance of 22 immune cells using transcriptome data [29].

### Tumor microenvironment (TME) and the risk score

The infiltration of stromal cells (also called stromal score), infiltration of immune cells (also called immune score) and tumor purity are considered important indicators of the TME, and these aspects were assessed via Estimation of Stromal and Immune cells in Malignant Tumor tissues using Expression data (ESTIMATE) [30]. Relationships between the ESTIMATE results and the risk score were then assessed. The scores of 13 specific stromal cells in BC patients from TCGA were also calculated by the xCell package of R software [31] and the relationships between the xCell results and the risk score were also evaluated. In addition, the connections between genes included in the risk signature and TME related parameters (including stromal cells, immune cells, stromal score, immune score and tumor purity) were also explored via the psych R package (the P value was corrected for multiple comparisons by the FDR method, and P. adj < 0.05 was considered statistically significant.). The above relevant results were visualized using the ggplot2 R package.

### The drug sensitivity and risk score

The connections between the half-maximal inhibitory concentration (IC50) of six common chemotherapy drugs (cisplatin, docetaxel, methotrexate, gemcitabine, paclitaxel and doxorubicin) and the risk score were explored using the pRRophetic R package [32]. As responses, including stable disease (SD), progressive disease (PD), complete response (CR) and partial response (PR), of each BC case to anti-PD-1 therapy were included in the IMvigor210 trial data, the effects of immunotherapy in the two risk groups were also analyzed.

### Functional assessment of the risk score

To explore the risk score-related biological functions, GSEA was performed. The “c2.cp.kegg.v7.4.symbols.gmt” gene set and “c5.all.v7.4.symbols.gmt” gene set of the MSigDB database were downloaded. NOM P value < 0.05 and FDR Q value < 0.25 were considered significant.

### Statistical analysis

The Wilcoxon rank sum test was used to compare the significant differences between the two groups. Log-rank analysis (survival curve) was utilized for survival analysis, correlation analysis was performed with the Spearman test, and the chi-square test was used to assess differences in the responses of the two groups of patients to immunotherapy. The non-parametric test was performed to compare differences between AUCs of the ROC curves also via the timeROC R package [33]. All statistical calculations were performed via R software (version 4.1.1).

## Results

### Preliminary exploration of HDAC9 in BC

**Fig 1:**
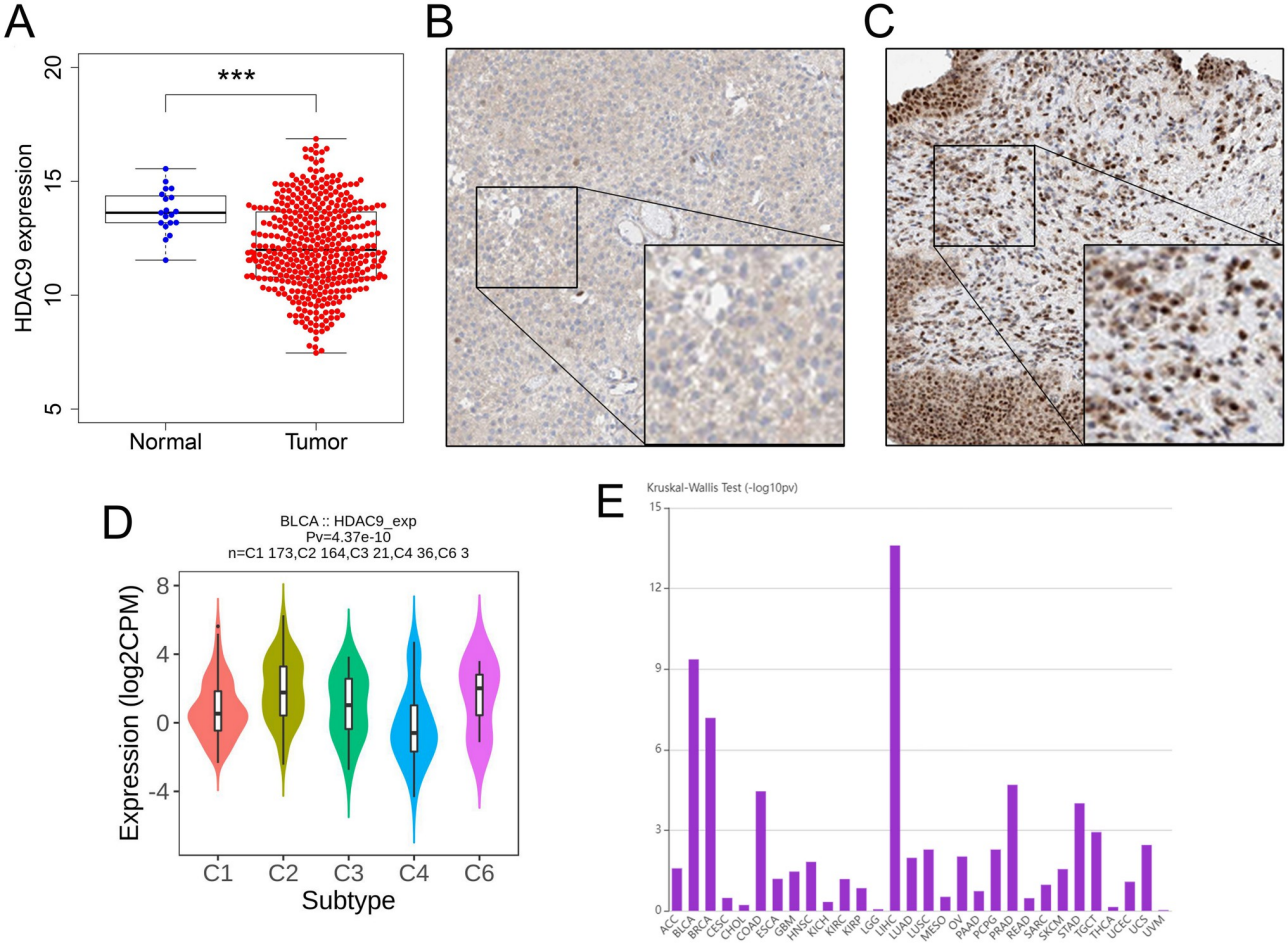
Preliminary exploration of HDAC9. According to TCGA data, low expression of HDAC9 was found in tumor samples (A). According to HPA data, lower expression of HDAC9 was found in tumor samples (B) than in normal samples (C). There was a significant difference between HDAC9 expression and immune subtype (D). It was worth noting that the significance level of the relationship between HDAC9 expression and immune subtypes in the BC ranked second among all tumors (E). HDAC9, histone deacetylase 9; TCGA, The Cancer Genome Atlas; HPA, Human Protein Atlas; BC, bladder cancer; C1, wound healing; C2, IFN-gamma dominant; C3, inflammatory; C4, lymphocyte depleted; C6, TGF-b dominant; ***, P < 0.001.

We included 411 tumor samples and 19 normal samples from TCGA, of which clinical information was available for 409 samples (Table 1). Compared with normal bladder tissues, HDAC9 was found to be lower in tumor tissues according to TCGA (Fig 1A) and HPA (Fig 1B and 1C) data. It was worth noting that the significance level of the relationship between HDAC9 expression and immune subtypes in the BC ranked second among all tumors (Fig 1D and 1E) [34]. HDAC9 was also closely related to various immunological characteristics [including tumor-infiltrating lymphocytes (TILs) (S1A–S1F Fig), immunosuppressive cytokines (S2A–S2F Fig), immune-activating cytokines (S3A–S3F Fig), and major histocompatibility complex (MHC) molecules (S4A–S4F Fig), all figures were downloaded directly from the TISIDB.]. NK T cells (TILs), PDCD1LG2 (immunosuppressive cytokines), ICOS (immune-activating cytokines) and HLA-DOB (MHC molecules) were the most positively correlated with HDAC9 expression.

**Table 1:** Characteristics of the BC patients obtained from the TCGA database.

Basic information		TCGA (n = 409)
Age		69 (median)
Gender	Female	106
	Male	303
Grade	High	385
	Low	21
	Unknow	3
Stage	I & II	132
	III & IV	275
	Unknow	2
T classification		124
	T3 & T4	253
	TX	1
	Unknow	31
N classification	N0	237
	N1 &N2 & N3	131
	NX	36
	Unknow	5
M classification	M0	194
	M1	11
	MX	202
	Unknow	2

BC, bladder cancer; TCGA, the The Cancer Genome Atlas.

### Establishment of the risk signature

**Fig 2:**
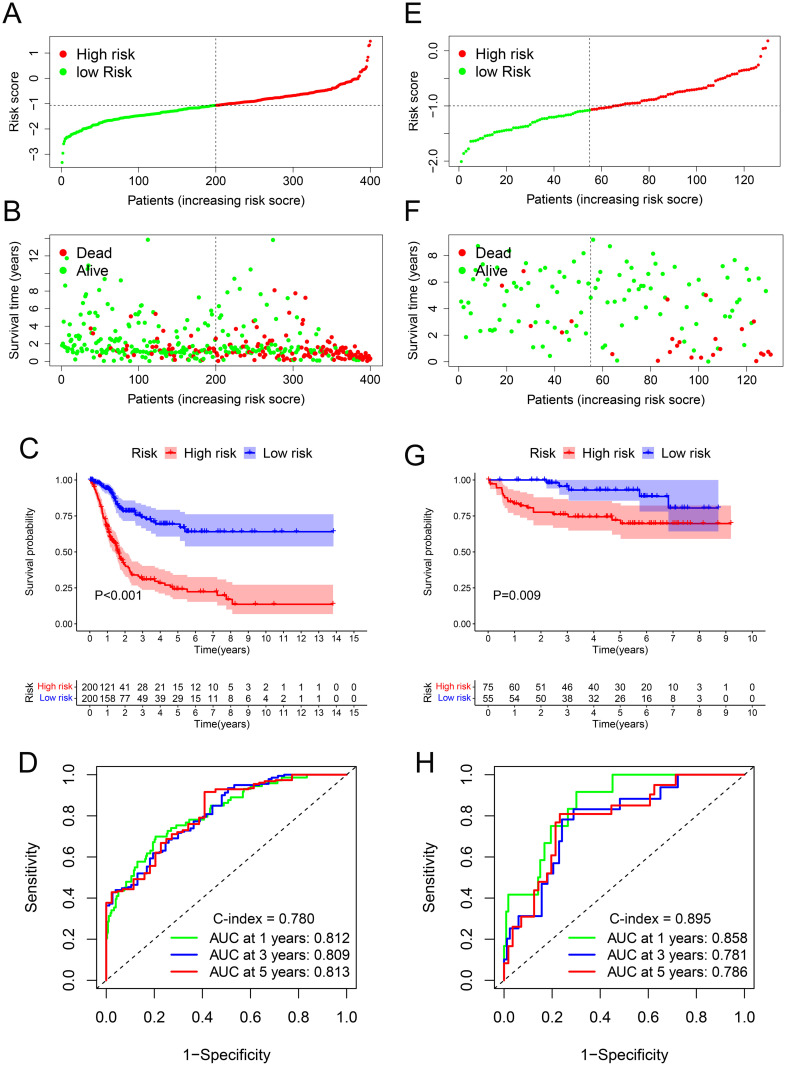
Establishment of the risk score. In the TCGA cohort, BC patients were distributed into different risk groups (A). Survival status of BC patients in different risk groups (B). The survival rate of the high-risk BC patients was worse than that of the low-risk patients (C). AUCs of the ROC curves were shown (D). In the verification cohort of the GEO, BC patients were distributed into different risk groups (E). Survival status of BC patients in different risk groups (F). The survival rate of the high-risk BC patients was worse than that of the low-risk patients (G). AUCs of the ROC curves were shown (H) TCGA, the Cancer Genome Atlas; GEO, Gene Expression Omnibus; AUC, area under the ROC curve; OS, overall survival; ROC, receiver operating characteristics; BC, bladder cancer.

According to the top five immune-related pathways in the GSEA results, HDAC9 played both immunosuppressive and immune activation effects in BC (Table 2). In total, 593 genes in the five pathways were extracted, and genes related to survival (P. adj < 0.05) were identified via univariable Cox regression (S1 Table). Then, through LASSO Cox regression, a risk score was calculated. (Fig 2A and 2B) (S2 Table). The survival rate of the high-risk BC patients was lower than that of the low-risk patients (P < 0.001) (Fig 2C). The areas under the curve (AUCs) were 0.812 (1 year), 0.809 (3 years) and 0.813 (5 years), and the C index was 0.780 (Fig 2D). The GSE32548 cohort, including 130 BC tissues and corresponding clinical data, was used to validate the signature (Fig 2E and 2F) (Table 3). The survival rate of the high-risk BC patients was also lower (P = 0.009) (Fig 2G), with AUCs of 0.858 (1 year), 0.781 (3 years) and 0.786 (5 years) and a C index of 0.895 (Fig 2H).

**Table 2:** Gene sets enriched in the high HDAC9 phenotype via GO.

Gene set name	NES	NOM *p*-val	FDR *q*-val
GO_NEGATIVE_REGULATION_OF_IMMUNE_SYSTEM_PROCESS	2.356	0.000	0.000
GO_POSITIVE_REGULATION_OF_IMMUNE_EFFECTOR_PROCESS	2.297	0.000	0.000
GO_NEGATIVE_REGULATION_OF_IMMUNE_EFFECTOR_PROCESS	2.275	0.000	0.000
GO_NEGATIVE_REGULATION_OF_IMMUNE_RESPONSE	2.275	0.000	0.000
GO_B_CELL_ACTIVATION_INVOLVED_IN_IMMUNE_RESPONSE	2.255	0.000	0.000

HDAC9, histone deacetylase 9; GO, Gene Ontology; NES: normalized enrichment score; NOM: nominal; FDR: false discovery rate.

Gene sets with NOM *p*-val < 0.05 and FDR *q*-val < 0.25 were considered significant.

**Table 3:** Characteristics of the BC patients obtained from the GEO database.

Basic information		GSE32548 (n = 130)
Age		70 (median)
Gender	Female	31
	Male	99
Grade	G1	15
	G2	40
	G3	75
T classification	< T2	91
	≥ T2	38
	TX	1

BC, bladder cancer; GEO, Gene Expression Omnibus.

**Fig 3:**
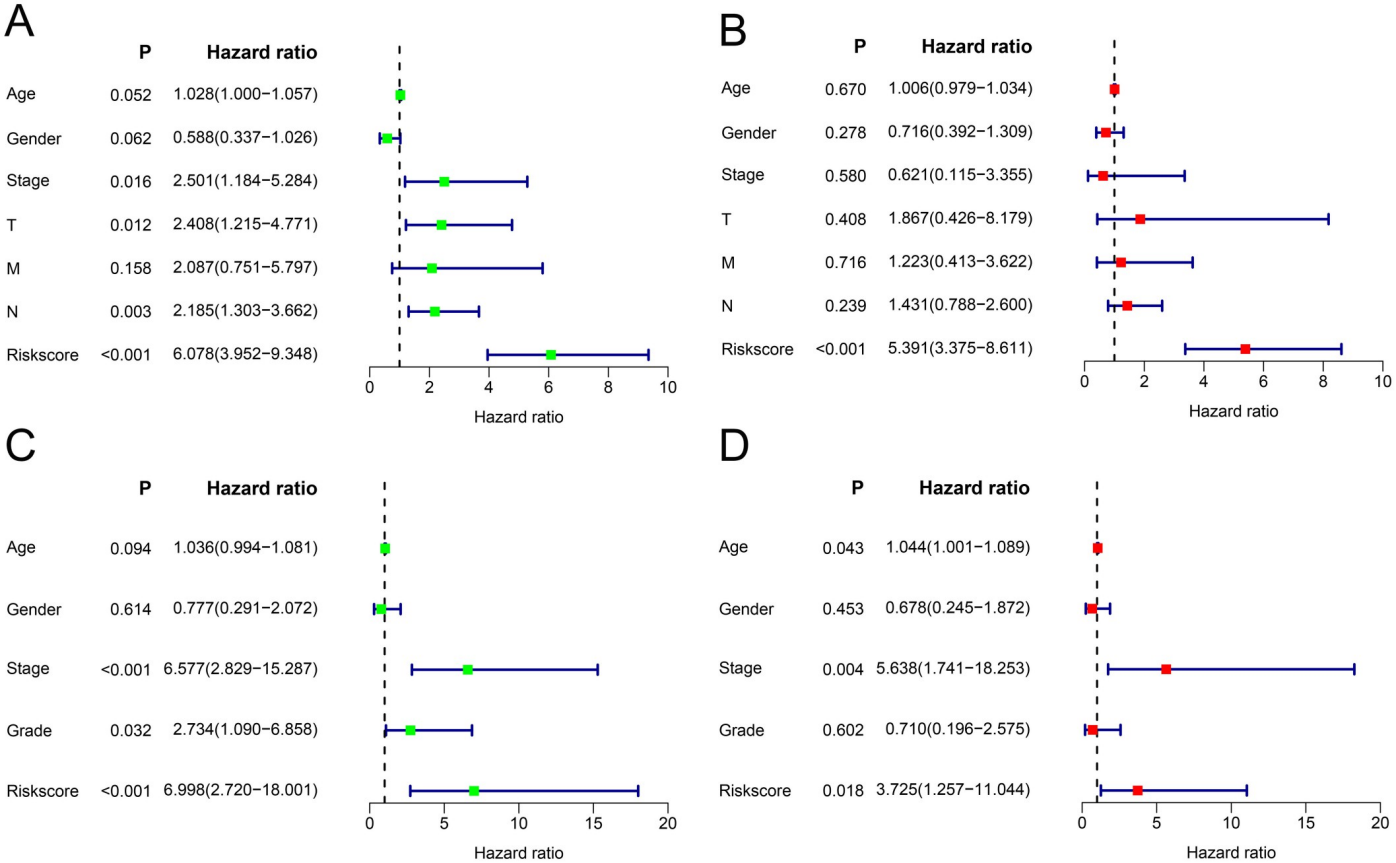
Independent prognostic analysis of the risk score. In the TCGA cohort, the results of univariate (A) and multivariate Cox analyses (B) showed that the risk score could be used as an independent prognostic factor for BC. In the GEO cohort, the results of univariate (C) and multivariate Cox analyses (D) also showed that the risk score could be used as an independent prognostic factor for BC. TCGA, The Cancer Genome Atlas; GEO, Gene Expression Omnibus; BC, bladder cancer.

**Fig 4:**
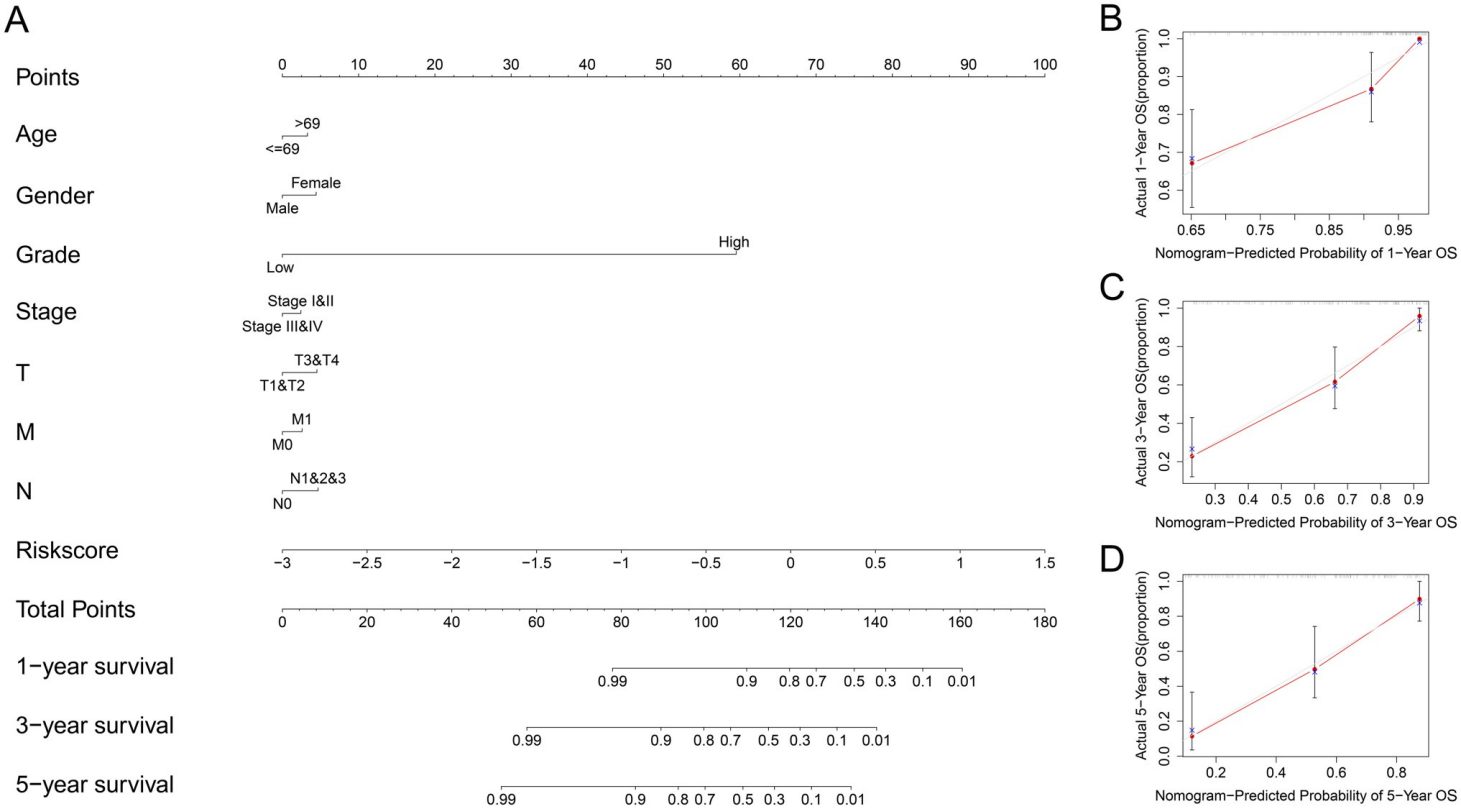
Establishment of the nomogram in TCGA. The results of the nomogram (A) and the calibration curve appropriately predicted 1-year (B), 3-year (C) and 5-year (D) OS for BC patients in TCGA. TCGA, The Cancer Genome Atlas; OS, overall survival; BC, bladder cancer.

**Fig 5:**
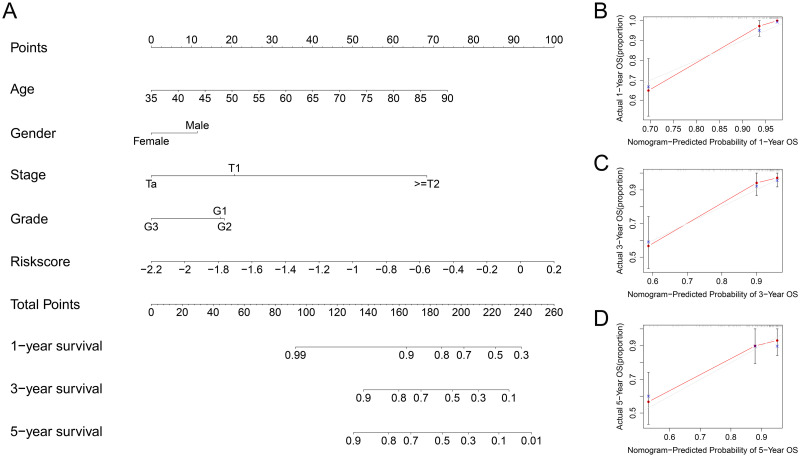
Establishment of the nomogram in GEO. The results of the nomogram (A) and the calibration curve appropriately predicted 1-year (B), 3-year (C) and 5-year (D) OS for BC patients in GEO. GEO, Gene Expression Omnibus; OS, overall survival; BC, bladder cancer.

**Fig 6:**
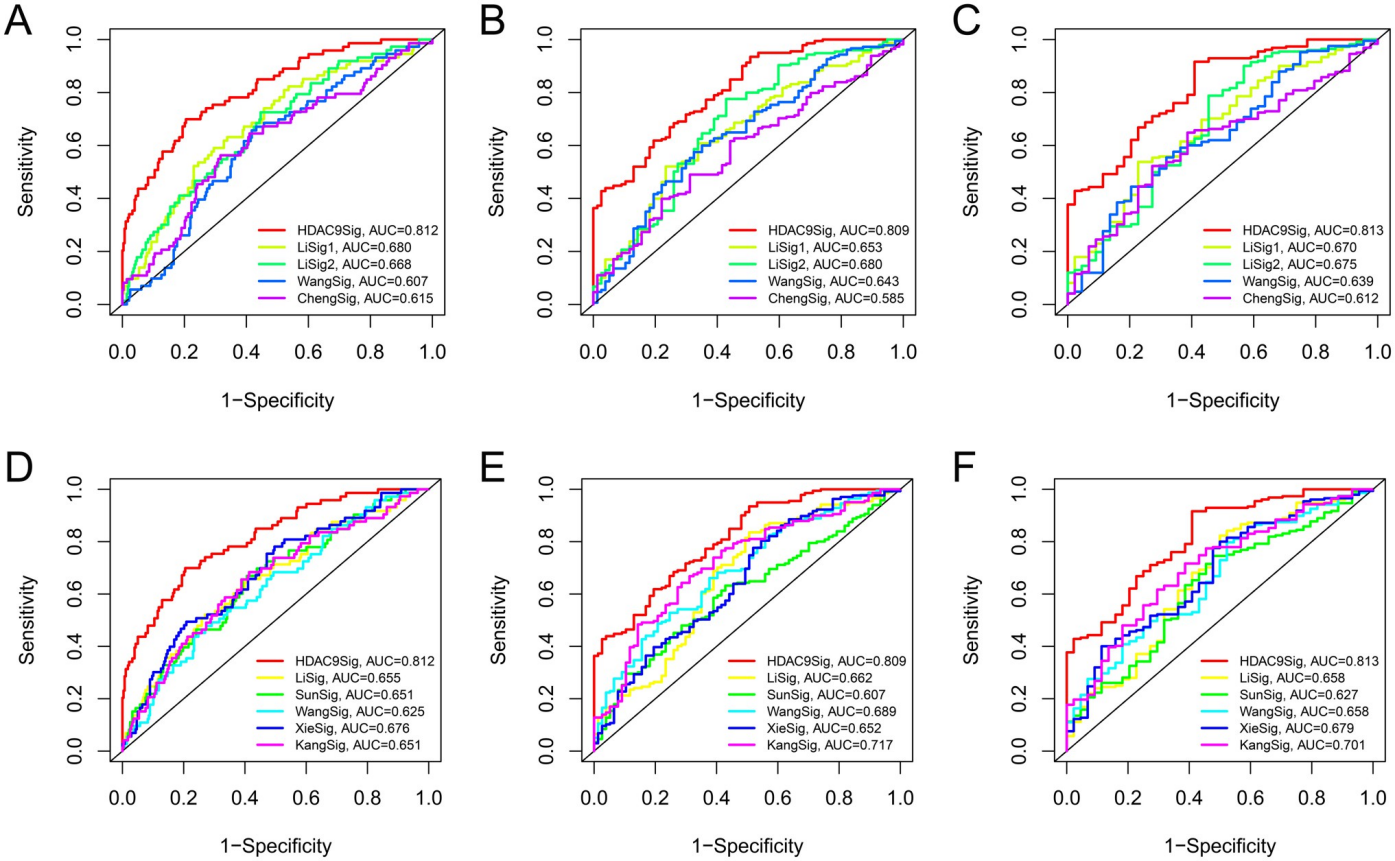
Comparison between signatures. By comparing with other signatures constructed through only prognostic-related genes (A-C) and other risk factors (D-F), our risk signature was found to be superior in performance.

In the TCGA cohort, univariate Cox regression suggested that clinical stage, T stage, N stage and the risk score were significantly related to poor prognosis (Fig 3A) (all P values < 0.05), and the risk score could be considered a prognostic factor based on multivariate Cox regression (Fig 3B). In the GSE32548 cohort, univariate Cox regression revealed that T stage, grade and risk score were closely related to poor prognosis in BC (all P values < 0.05) (Fig 3C), and the risk score was also identified as a prognostic factor based on multivariate Cox regression (Fig 3D). Additionally, the nomogram and calibration curves appropriately predicted 1-year, 3-year and 5-year OS for both TCGA (Fig 4A–4D) and GSE32548 (Fig 5A–5D). Upon comparison with other signatures constructed through only prognosis-related genes (Fig 6A–6C) or other risk factors (Fig 6D–6F), our risk signature was found to be superior (Tables 4 and 5).

**Table 4:** Comparison of ROC curve AUCs between HDAC9-associated immune-related signature and risk signatures based only on prognostic-related genes.

AUCs	1 year (P value)	3 years (P value)	5 years (P value)
HDAC9Sig vs LiSig1	0.001	0.000	0.002
HDAC9Sig vs LiSig2	0.001	0.003	0.017
HDAC9Sig vs WangSig	0.000	0.000	0.000
HDAC9Sig vs ChengSig	0.000	0.000	0.000

ROC, receiver operating characteristics; AUC, area under the curve; HDAC9, histone deacetylase 9; Sig, signature.

**Table 5:** Comparison of ROC curve AUCs between HDAC9-associated immune-related signature and other risk signatures based on other risk factors.

AUCs	1 year (P value)	3 years (P value)	5 years (P value)
HDAC9Sig vs LiSig	0.000	0.000	0.003
HDAC9Sig vs SunSig	0.000	0.000	0.000
HDAC9Sig vs WangSig	0.000	0.002	0.001
HDAC9Sig vs XieSig	0.000	0.000	0.001
HDAC9Sig vs KangSig	0.000	0.012	0.013

ROC, receiver operating characteristics; AUC, area under the curve; HDAC9, histone deacetylase 9; Sig, signature.

The relationships between HDAC9 and selected genes were assessed by coexpression analysis (S5 Fig). CDK6 was identified as the most positively correlated with HDAC9 expression, whereas NR1H2 was the most negatively correlated with HDAC9 expression.

### The risk score was related to infiltration of various immune cells

**Fig 7:**
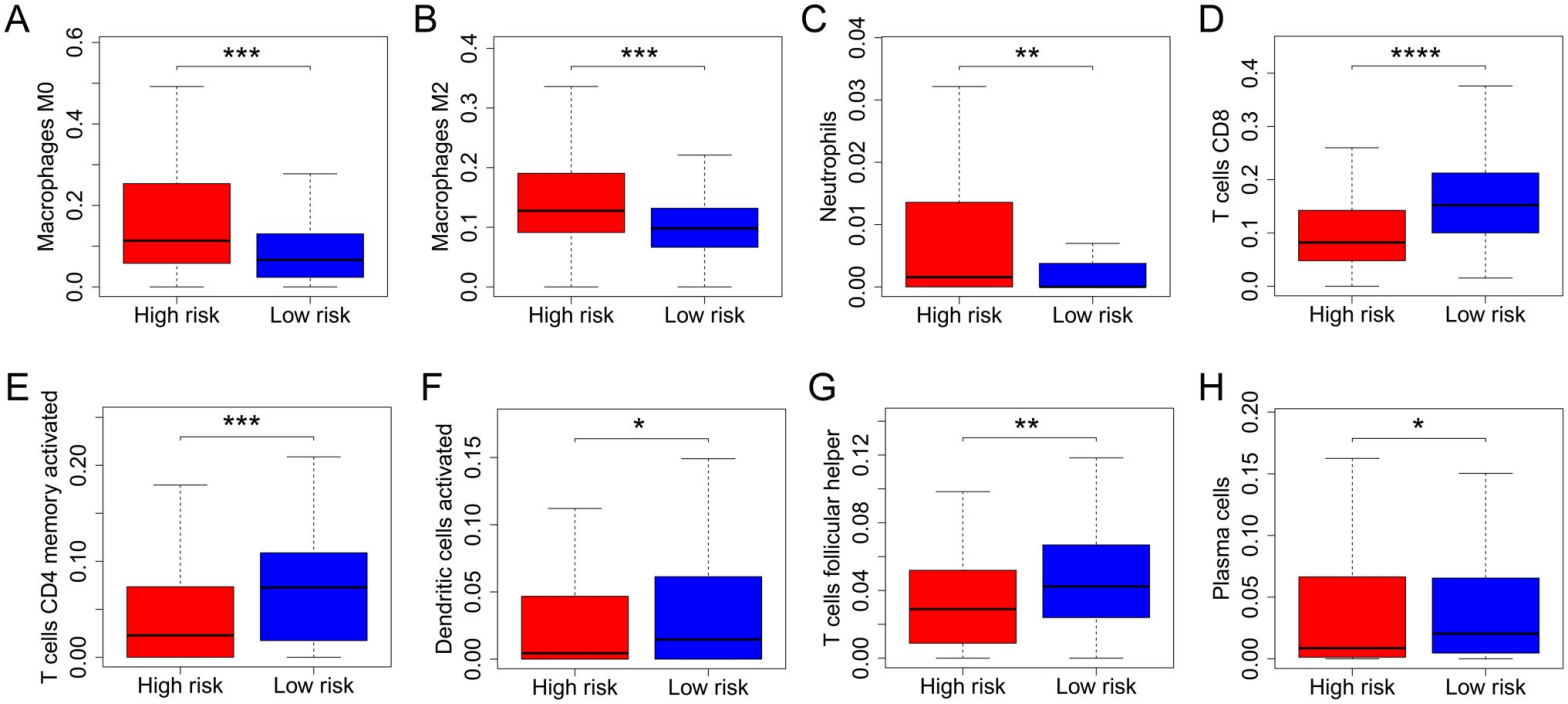
The risk score was associated with infiltration of various immune cells. The results of CIBERSORT indicated enrichment of signatures of M0 (A) and M2 macrophages (B) and neutrophils (C) in high-risk BC patients. Conversely, signatures of CD8 T cells (D), CD4 memory activated T cells (E), activated dendritic cells (F), follicular helper T cells (G) and plasma cells (H) were enriched in low-risk BC patients. CIBERSORT, cell type identification by estimating relative subsets of RNA transcripts; BC, bladder cancer; *, P <0.05; **, P < 0.01; ***, P < 0.001.

The results of CIBERSORT revealed enrichment for signatures of M0 (Fig 7A) and M2 macrophages (Fig 7B) and neutrophils (Fig 7C) in high-risk BC patients (all P values < 0.05). Conversely, signatures of CD8 T cells (Fig 7D), activated memory CD4 T cells (Fig 7E), activated dendritic cells (Fig 7F), follicular helper T cells (Fig 7G) and plasma cells (Fig 7H) were enriched in low-risk BC patients (all P values < 0.05).

### The risk score could affect the change of TME

**Fig 8:**
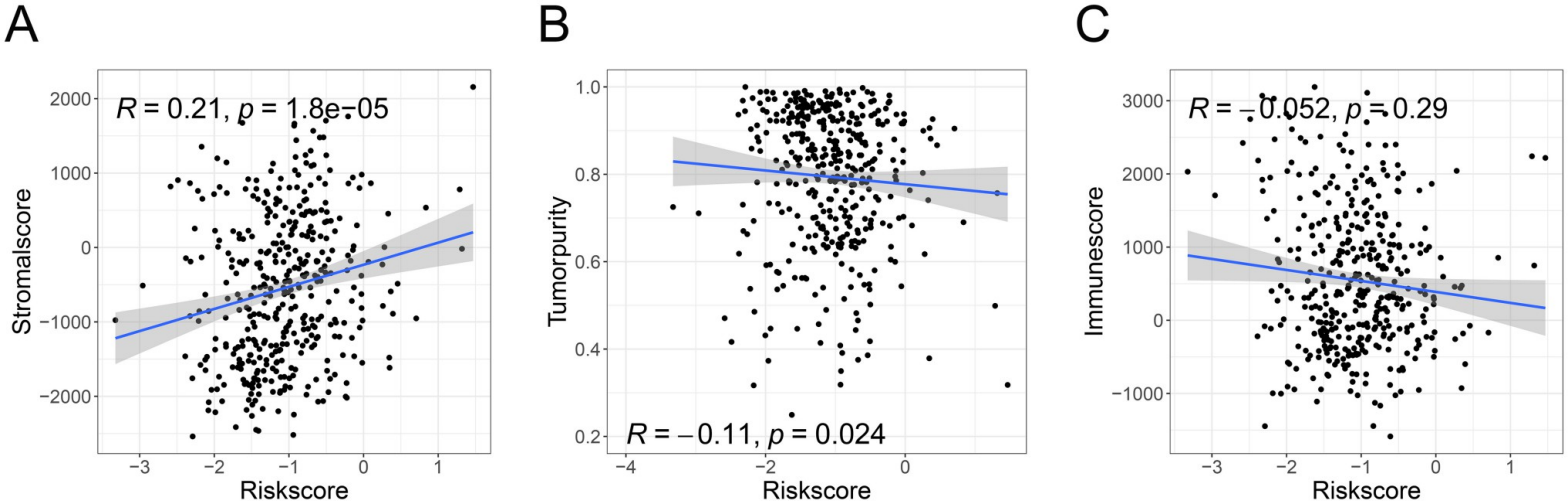
The risk score was significantly related to the TME based on the ESTIMATE algorithm. The stromal score was positively correlated with the risk score (A), and tumor purity was negatively correlated with the risk score (B). There was no significant difference between the risk score and immune score (C). TME, tumor microenvironment.

**Fig 9:**
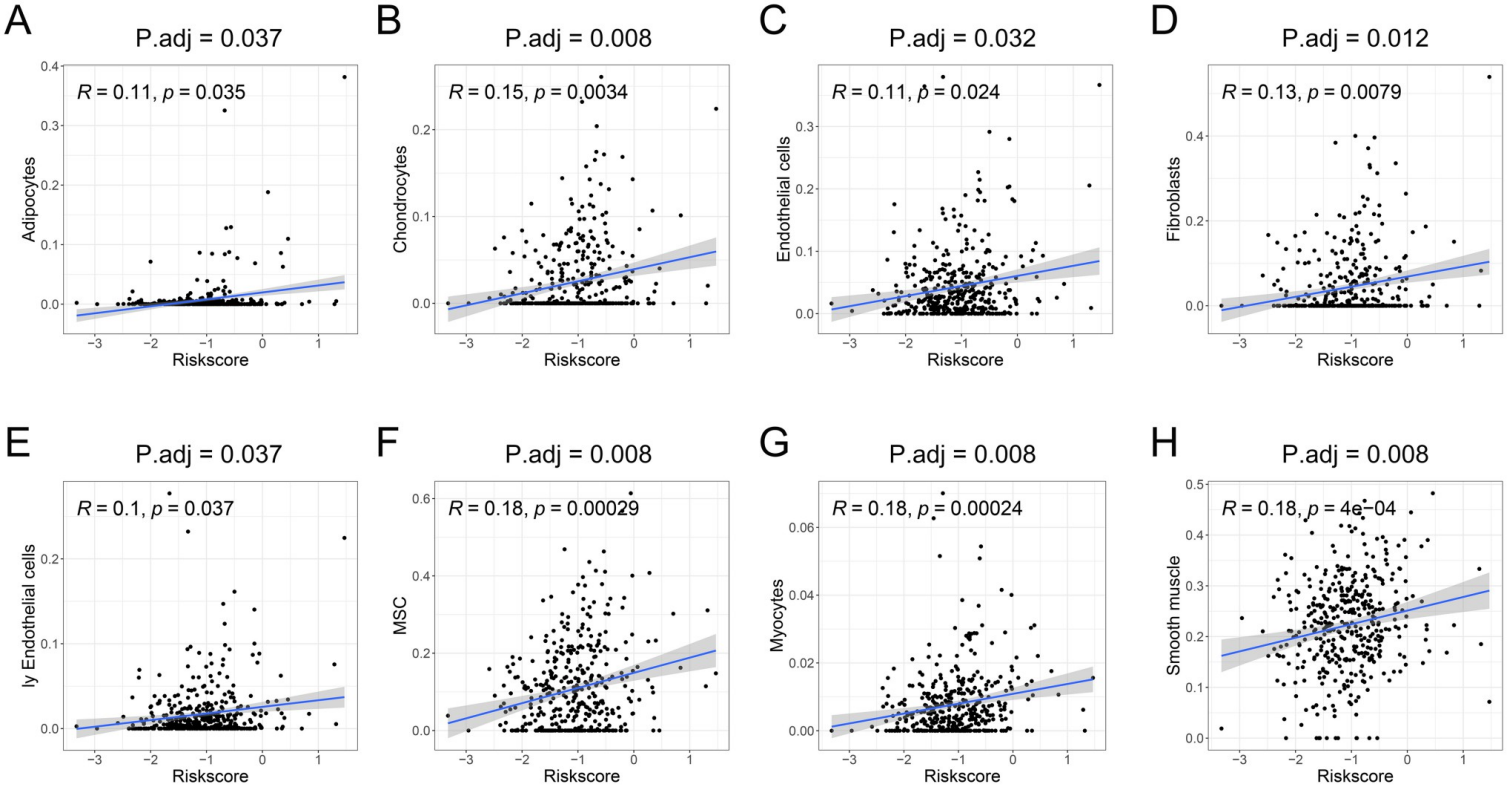
Stromal cells in BC patients based on the xCell algorithm. Results indicated that enrichment of signatures of eight stromal cells [adipocytes (A), chondrocytes (B), endothelial cells (C), fibroblasts (D), ly endothelial cells (E), MSCs (F), myocytes (G) and smooth muscle (H)] from the TME in the high-risk group. BC, bladder cancer; MSCs, mesenchymal stem cells; ly endothelial cells, lymphatic endothelial cells; TME, tumor microenvironment; P. adj, P. adjust.

The relationship between the TME (including stromal score, immune score and tumor purity) and the risk score was also evaluated. The results revealed that the stromal score was positively correlated with the risk score (Fig 8A), but tumor purity was negatively correlated (Fig 8B) (all P values < 0.05). Nevertheless, there was no significant difference between the immune score and risk score (Fig 8C). In addition, signatures of adipocytes (Fig 9A), chondrocytes (Fig 9B), endothelial cells (Fig 9C), fibroblasts (Fig 9D), lymphatic endothelial cells (ly endothelial cells) (Fig 9E), mesenchymal stem cells (MSCs) (Fig 9F), myocytes (Fig 9G), and smooth muscle (Fig 9H) were enriched in high-risk BC patients (all P. adj < 0.05). Besides, the connections between genes included in the risk signature and TME related parameters (including stromal cells, immune cells, stromal score, immune score and tumor purity) were also explored and visualized via a heatmap (S6 Fig).

### Risk score and drug sensitivity

**Fig 10:**
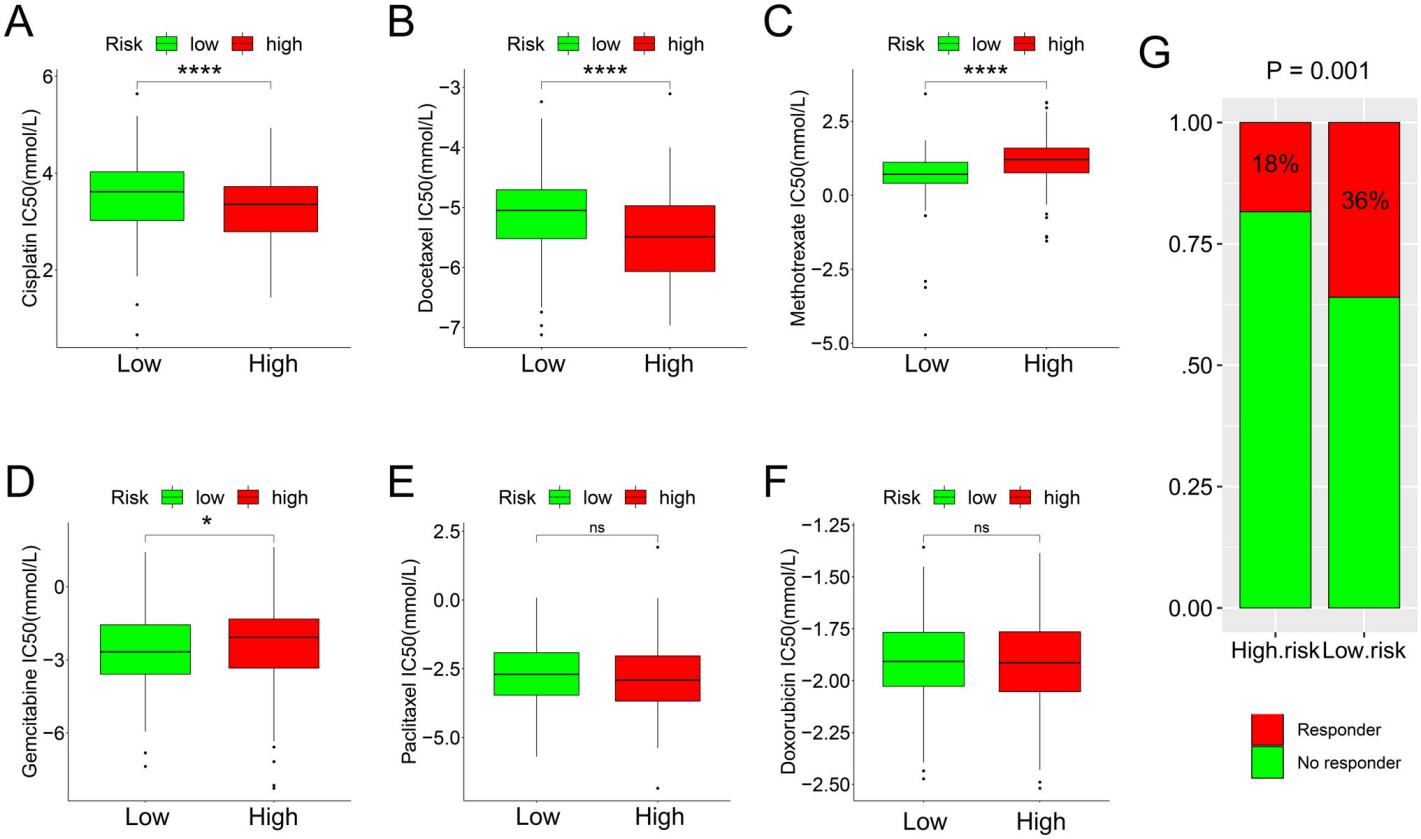
Risk score and drug sensitivity. Cisplatin (A) and docetaxel (B) exhibited higher IC50 values in low-risk patients; thus, the high-risk group was more sensitive to cisplatin and docetaxel. Methotrexate (C) and gemcitabine (D) exhibited a higher IC50 in high-risk BC patients; thus, low-risk BC patients were more sensitive to methotrexate and gemcitabine. However, there were no significant differences between other chemotherapy drugs (paclitaxel and doxorubicin) and the risk score (E-F). The CR/PR rate in low-risk patients was higher than that in high-risk patients (G), indicating more significant immunotherapy benefits for low-risk patients. IC50, half-maximal inhibitory concentration; SD, stable disease; PD, progressive disease; CR, complete response; PR, partial response; BC, bladder cancer; *, P <0.05; ****, P < 0.0001.

We predicted the IC50 of six common chemotherapy drugs in the different groups. Cisplatin (Fig 10A) and docetaxel (Fig 10B) exhibited higher IC50 values in low-risk patients; thus, the high-risk group was more sensitive to cisplatin and docetaxel (all P < 0.05). Methotrexate (Fig 10C) and gemcitabine (Fig 10D) exhibited a higher IC50 in high-risk BC patients; thus, low-risk BC patients were more sensitive to methotrexate and gemcitabine (all P < 0.05). However, there were no significant differences between other chemotherapy drugs (paclitaxel and doxorubicin) and the risk score (Fig 10E and 10F). By analyzing data from the IMvigor210 trial (including 68 CR/PR patients and 230 SD/PD patients), we also found that the CR/PR rate in low-risk patients was higher than that in high-risk patients, which suggested that low-risk patients benefit significantly more from immunotherapy than high-risk patients (Fig 10G).

### Analysis of biological functions

The biological functions of the risk score were evaluated via GSEA. The most significant biofunctions enriched in the high-risk group based on Gene Ontology (GO) and Kyoto Encyclopedia of Genes and Genomes (KEGG) analyses are listed in Tables 6 and 7, respectively. The most significant biofunctions enriched in the low-risk group based on KEGG analysis are listed in Table 8. Unfortunately, no significant pathways were found to be enriched in low-risk BC patients based on GO analyses.

**Table 6:** Gene sets enriched in the high-risk phenotype via GO.

Gene set name	NES	NOM *p*-val	FDR *q*-val
GO_REGULATION_OF_CHONDROCYTE_DIFFERENTIATION	2.137	0.002	0.033
GO_SMOOTH_MUSCLE_CONTRACTION	2.131	0.000	0.030
GO_CONNECTIVE_TISSUE_DEVELOPMENT	2.115	0.000	0.029
GO_CELL_CELL_JUNCTION_ASSEMBLY	2.102	0.000	0.029
GO_POSITIVE_REGULATION_OF_CELL_DIVISION	2.099	0.000	0.029
GO_CELL_CELL_JUNCTION	2.095	0.000	0.030
GO_WNT_PROTEIN_BINDING	2.074	0.000	0.033
GO_TUBULIN_BINDING	2.059	0.000	0.029
GO_EPITHELIAL_TO_MESENCHYMAL_TRANSITION	2.034	0.000	0.034
GO_CHONDROCYTE_DEVELOPMENT	1.923	0.002	0.043
GO_FOLIC_ACID_METABOLIC_PROCESS	1.962	0.002	0.036
GO_POSITIVE_REGULATION_OF_ENDOTHELIAL_CELL_PROLIFERATION	1.916	0.004	0.044
GO_REGULATION_OF_GLUCOSE_TRANSMEMBRANE_TRANSPORT	1.905	0.004	0.044
GO_POSITIVE_REGULATION_OF_FIBROBLAST_MIGRATION	1.783	0.010	0.058
GO_ACTIVATION_OF_MAPK_ACTIVITY	1.762	0.026	0.061
GO_REGULATION_OF_STEROID_METABOLIC_PROCESS	1.733	0.006	0.068
GO_POSITIVE_REGULATION_OF_DNA_REPLICATION	1.694	0.048	0.076
GOBP_NEGATIVE_REGULATION_OF_CELL_CYCLE_G1_S_PHASE_TRANSITION	1.685	0.025	0.078
GO_CELLULAR_GLUCOSE_HOMEOSTASIS	1.656	0.000	0.084
GO_REGULATION_OF_LIPID_METABOLIC_PROCESS	1.638	0.041	0.086

GO, Gene Ontology; NES: normalized enrichment score; NOM: nominal; FDR: false discovery rate.

Gene sets with NOM *p*-val < 0.05 and FDR *q*-val < 0.25 were considered significant.

**Table 7:** Gene sets enriched in the high-risk phenotype via KEGG.

Gene set name	NES	NOM *p*-val	FDR *q*-val
KEGG_ADHERENS_JUNCTION	2.069	0.002	0.012
KEGG_FOCAL_ADHESION	2.023	0.002	0.014
KEGG_ECM_RECEPTOR_INTERACTION	1.987	0.006	0.014
KEGG_TGF_BETA_SIGNALING_PATHWAY	1.941	0.000	0.019
KEGG_CELL_CYCLE	1.891	0.025	0.021
KEGG_WNT_SIGNALING_PATHWAY	2.090	0.000	0.023
KEGG_STEROID_BIOSYNTHESIS	1.819	0.016	0.040
KEGG_PURINE_METABOLISM	1.795	0.004	0.047
KEGG_VASCULAR_SMOOTH_MUSCLE_CONTRACTION	1.749	0.015	0.053
KEGG_GLYCOSAMINOGLYCAN_BIOSYNTHESIS_CHONDROITIN_SULFATE	1.696	0.036	0.062
KEGG_CYSTEINE_AND_METHIONINE_METABOLISM	1.644	0.039	0.077
KEGG_GLYCOLYSIS_GLUCONEOGENESIS	1.615	0.026	0.085
KEGG_MAPK_SIGNALING_PATHWAY	1.619	0.021	0.087
KEGG_SPHINGOLIPID_METABOLISM	1.558	0.045	0.101
KEGG_BLADDER_CANCER	1.543	0.036	0.108

KEGG, Kyoto Encyclopedia of Genes and Genomes; NES: normalized enrichment score; NOM: nominal; FDR: false discovery rate.

Gene sets with NOM *p*-val < 0.05 and FDR *q*-val < 0.25 were considered significant.

**Table 8:** Gene sets enriched in the low-risk phenotype via KEGG.

ID	NES	NOM *p*-val	FDR *q*-val
KEGG_LINOLEIC_ACID_METABOLISM	-1.902	0.002	0.210
KEGG_RIG_I_LIKE_RECEPTOR_SIGNALING_PATHWAY	-1.841	0.012	0.179
KEGG_ARACHIDONIC_ACID_METABOLISM	-1.632	0.017	0.206

KEGG, Kyoto Encyclopedia of Genes and Genomes; NES: normalized enrichment score; NOM: nominal; FDR: false discovery rate.

Gene sets with NOM *p*-val < 0.05 and FDR *q*-val < 0.25 were considered significant.

## Discussion

Currently, metabolic reprogramming, immune evasion and tumor-promoting inflammation are the three hallmarks of cancers [35]. As members of histone deacetylation metabolism, the role of HDACs in tumors has received widespread attention. As the HDAC IIa subtype, HDAC9 was found to play a dual role in tumors, which might be associated with the content of target proteins, alternative splicing, transcription factors that bind to the HDAC9 N-terminal region, and phosphorylation differences in multiple tumors [11, 36–39].

HDAC9 was initially found to be closely related to a variety of immunological parameters. And it was differentially expressed in five immune subtypes (previous research suggested that six immune subtypes could be identified in tumors, and there were significant differences in immune infiltration and sensitivity to immunotherapy among subtypes, five immune subtypes could be identified in BC) [34], implying that HDAC9 could be used to classify immune subtypes, which would help individualized treatment. However, further follow-up work is required to improve these results.

In our research, genes related to survival were identified from the top five enriched immune-related pathways based on the high expression status of HDAC9. Then, a risk signature using the selected genes was constructed. The high-risk group appeared to have a worse prognosis and be more sensitive to cisplatin and docetaxel. In contrast, low-risk BC patients were more sensitive to methotrexate and gemcitabine. Furthermore, low-risk patients had more significant benefits from immunotherapy.

To date, six genes in the risk signature have been studied in relation to prognosis or TME in BC. Overexpression of LGALS1, MMP28, RNF26 and PHB could lead to poor clinical outcome of BC [40–43]. Increased KLRK1 expression was associated with better prognosis and was positively related to the activation of NK cells [44]. Decreased expression of PTPN6 suggested a poor prognosis and was connected with the infiltration of a variety of immune cells [45]. Interestingly, among the genes included in the risk signature, most were involved in metabolic processes. ANXA1, BCL6, CDK6, CLEC12B, FBXO7, FGR, GBP1, IRAK3, MMP28, NPLOC4, PTPN6, PTPRJ, RNF26, SUPT6H, TRIM27 and ZC3H8 participated in protein metabolism; BCL6, ZC3H8 and SUPT6H acted as transcriptional regulators [46–48]; MMP28 is a proteolysis factor [49]; ANXA1, CDK6, CLEC12B, FBXO7, FGR, GBP1, IRAK3, NPLOC4, PTPN6, PTPRJ, RNF26 and TRIM27 were involved in the posttranslational modification of proteins [45, 50–60]; ADIPOQ, ANGPT1, IL21, LDLR, OTOP1, ZBTB7B and ZC3H12A were closely related to lipid metabolism [61–67]; GPR68, IL12A, INS, KLRK1 and LGALS1 were found to regulate glucose metabolism [68–72]; MSH6 and TP53BP1 affected nucleotide metabolism by regulating DNA repair [73, 74]; and NR1H2 and PHB could simultaneously influence both glucose and lipid metabolism [75, 76]. Although immune-related pathways were activated under high HDAC9 expression, most enriched genes were metabolically related. In other words, changes in HDAC9, a regulator of protein posttranslational modifications, could potentially be connected to metabolic disorders. Metabolic disorders might lead to changes in HDAC9 expression and activity, or metabolism and HDAC9 could both be regulated by the same upstream signal; this will require rigorous biological experiments to verify. Therefore, immunity and metabolism were closely linked in BC. By observing the coexpression of HDAC9 and the genes included in the risk signature and the degree of difference, more potential factors could be selected for subsequent research.

By comparing with other risk signatures constructed only through prognosis-related genes, our risk signature was found to be superior in performance. One reason for this could be that the signature constructed by only prognosis-related genes was mixed with numerous factors that affect the prognosis of tumors (such as hypoxia and inflammation). The risk signature in our research used a metabolic factor (HDAC9) for stratification, and most of the immune-related genes included in the risk signature were related to various metabolic processes, which effectively reduced confounding factors. We focused on the influence of metabolic processes on BC. Interestingly, by comparing BC signatures constructed with other risk factors, the signature we built still had advantages, which indicated that the metabolic process in BC should attract attention.

The connection between the risk score and TME was further analyzed. The ESTIMATE results showed that the risk score was positively correlated with the stromal score and negatively correlated with tumor purity, and there was no significant difference between the risk score and the immune score. However, the evaluation of specific immune cells through the CIBERSORT algorithm suggested that M0 and M2 macrophages and neutrophils were more enriched in the high-risk group, whereas CD8 T cells, activated memory CD4 T cells, follicular helper T cells, activated dendritic cells and plasma cells were more enriched in the low-risk group. M0 and M2 macrophages were positively correlated with a poor prognosis of BC [77, 78], while a higher infiltration level of CD8 T cells was correlated with a good prognosis [79, 80]. Therefore, the constructed risk score could appropriately assess immune cell infiltration, and it had an impact on immune cell infiltration. The lack of an association between overall immune score and risk score based on the ESTIMATE results suggested that the accumulation of negative effector immune cells in the high-risk group was close to that of positive effector immune cells in the low-risk group, resulting in no significant differences between the two groups. Additionally, the stromal score, which was significantly positively correlated with the risk score, could indicate that elevated stromal cells play an important role in different risk groups.

Through further analysis, enrichment of signatures of adipocytes, chondrocytes, endothelial cells, fibroblasts, ly endothelial cells, MSCs, myocytes, and smooth muscle was found in the high-risk BC patients. Studies have shown that adipocytes promote breast cancer metastasis [81] and that chondrocyte can promote the progression of tumors by secreting inflammatory factors [82]. By combining the results of ESTIMATE analysis, we hypothesize that the decrease in tumor purity due to the increased stromal cell infiltration might contribute to the poor prognosis of high-risk patients.

Chemotherapy is an important treatment for BC, and cisplatin is considered to be the first-line chemotherapy for advanced BC [83]. However, due to drug toxicity and heterogeneity among patients, 40–60% of patients with bladder cancer do not respond to cisplatin-based systemic chemotherapy [84, 85]. Studies have pointed out that the drug sensitivity of cisplatin in BC is closely related to ERKs and androgen receptors [86, 87]. Despite the existence of various cancer treatment strategies, chemotherapy drugs are still widely used. Due to drug resistance and side effects, the therapeutic effects of chemotherapeutic drugs are often very different and difficult to predict, and the risk score calculated by various related risk factors could be significantly related to drug sensitivity [88]. The risk signature we built could develop personalized treatments by stratifying the risks of BC patients. Adjusting the drug dosage and choosing anticancer treatment strategies according to individual specificities could improve the prognosis of patients and minimize side effects. The six chemotherapy drugs included in the study affected the metabolic process to varying degrees. Among the chemotherapeutic drugs with differences between the two risk groups, cisplatin could induce cell cycle G1/S arrest [89], docetaxel promoted the formation of tubulin structures [90], methotrexate prohibited folic acid metabolism [91] and gemcitabine inhibited DNA replication [92]. The GO results indicated G1/S cell cycle arrest (GOBP_NEGATIVE_REGULATION_OF_CELL_CYCLE_G1_S_PHASE_TRANSITION), tubulin structure synthesis (GO_TUBULIN_BINDING), folic acid metabolism process (GO_FOLIC_ACID_METABOLIC_PROCESS), and the DNA replication (GO_POSITIVE_REGULATION_OF_DNA_REPLICATION) related biological functions were enriched in the high-risk group, implying that chemotherapy effectiveness depended on whether the drug mechanism conflicted with the metabolic changes of the patients themselves. If they conflicted (such as methotrexate and gemcitabine), the effectiveness would be reduced, and vice versa (such as cisplatin and docetaxel). Chemotherapeutic drugs with similar mechanisms may mainly act on different metabolic changes in patients selectively, which might eventually lead to the opposite results. These should be the content of subsequent in vivo and in vitro precise pharmacological experiments.

GSEA was conducted to explore biological mechanisms associated with the risk score. Based on GO analysis, a large number of stromal cells, junctions between cells and metabolic process-related biofunctions were significantly enriched in the high-risk BC patients. The results of KEGG revealed extracellular matrix (ECM), metabolic process and common BC-related pathways (WNT, TGF-β and MAPK pathways) to be enriched in the high-risk phenotype [93–98]. The ECM is crucial for maintaining tissue homeostasis, and its disruption can promote tumor occurrence, progression, and metastasis by inducing EMT [99–103]. Hence, the GO, KEGG, ESTIMATE and xCell results were consistent.

Finally, our research has some limitations. First, this research was retrospective in design, and due to the comparison of patients from different cohorts, heterogeneity existed. Therefore, it is necessary to verify the prospective cohort used in the future. Second, the results of GSEA stratified by HDAC9 status and TISIDB suggested that HDAC9 could play a dual role in immune function, and further analysis should be conducted to clarify the net effect of HDAC9 on immune function and related mechanisms in future studies. Third, the inherent molecular mechanism of the risk signature affecting the sensitivity of tumor chemotherapy drugs is worthy of in-depth exploration.

In summary, an immune-related prognostic signature based on HDAC9 expression that could independently predict BC patient prognosis was constructed. The genes included in this risk score may constitute new targets for the treatment of BC, and further study is warranted. Moreover, the use of this signature will help clinicians make personalized treatment decisions.

## Supporting information

S1 Fig The relationship between HDAC9 expression and TILs. The first six TILs with the strongest correlation with HDAC9 in BC were visualized (A-F). HDAC9, histone deacetylase 9; TILs, tumor-infiltrating lymphocytes; BC, bladder cancer.
(TIF)

S2 Fig The relationship between HDAC9 expression and immunosuppressive cytokines. The first six immunosuppressive cytokines with the strongest correlation with HDAC9 in BC were visualized (A-F). HDAC9, histone deacetylase 9; BC, bladder cancer.
(TIF)

S3 Fig The relationship between HDAC9 expression and immune-activating cytokines. The first six immune-activating cytokines with the strongest correlation with HDAC9 in BC were visualized (A-F). HDAC9, histone deacetylase 9; BC, bladder cancer.
(TIF)

S4 Fig The relationship between HDAC9 expression and MHC molecules. The first six immune-activating cytokines with the strongest correlation with HDAC9 in BC were visualized (A-F). HDAC9, histone deacetylase 9; MHC, major histocompatibility complex; BC, bladder cancer.
(TIF)

S5 Fig Coexpression analysis. Coexpression analysis was performed to further understand connections between HDAC9 and the risk score genes. The relevant results were visualized via a heatmap. HDAC9, histone deacetylase 9; *, P. adjust <0.05; **, P. adjust < 0.01.
(TIF)

S6 Fig The relationship between genes included in the risk signature and TME. Correlation analysis was performed to further understand connections between genes included in the risk signature and TME. The relevant results were visualized via a heatmap. TME, tumor microenvironment; *, P. adjust <0.05; **, P adjust < 0.01.
(TIF)

S1 Table Genes related to survival were distinguished via univariable Cox regression. (DOCX)

S2 Table The coefficients of included genes. (DOCX)
