# Supplementary figures and images for: An HDAC9-associated immune-related signature predicts bladder cancer prognosis
Source: PLoS One. 2022 Mar 3;17(3):e0264527. doi: 10.1371/journal.pone.0264527 (PMC8893690; doi:10.1371/journal.pone.0264527)

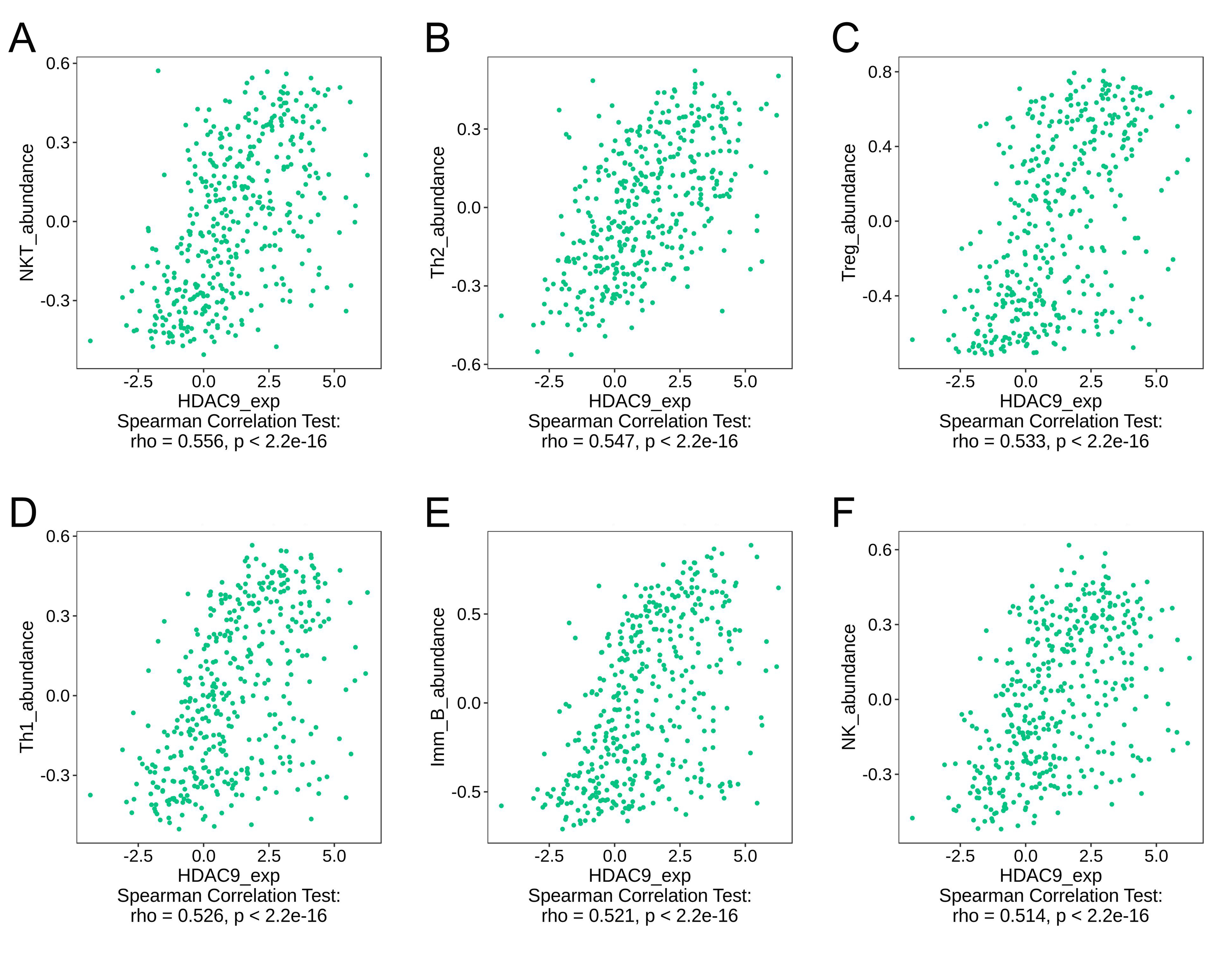

Supplement: S1 Fig — The first six TILs with the strongest correlation with HDAC9 in BC were visualized (A-F). HDAC9, histone deacetylase 9; TILs, tumor-infiltrating lymphocytes; BC, bladder cancer. (TIF) [file pone.0264527.s001.tif]

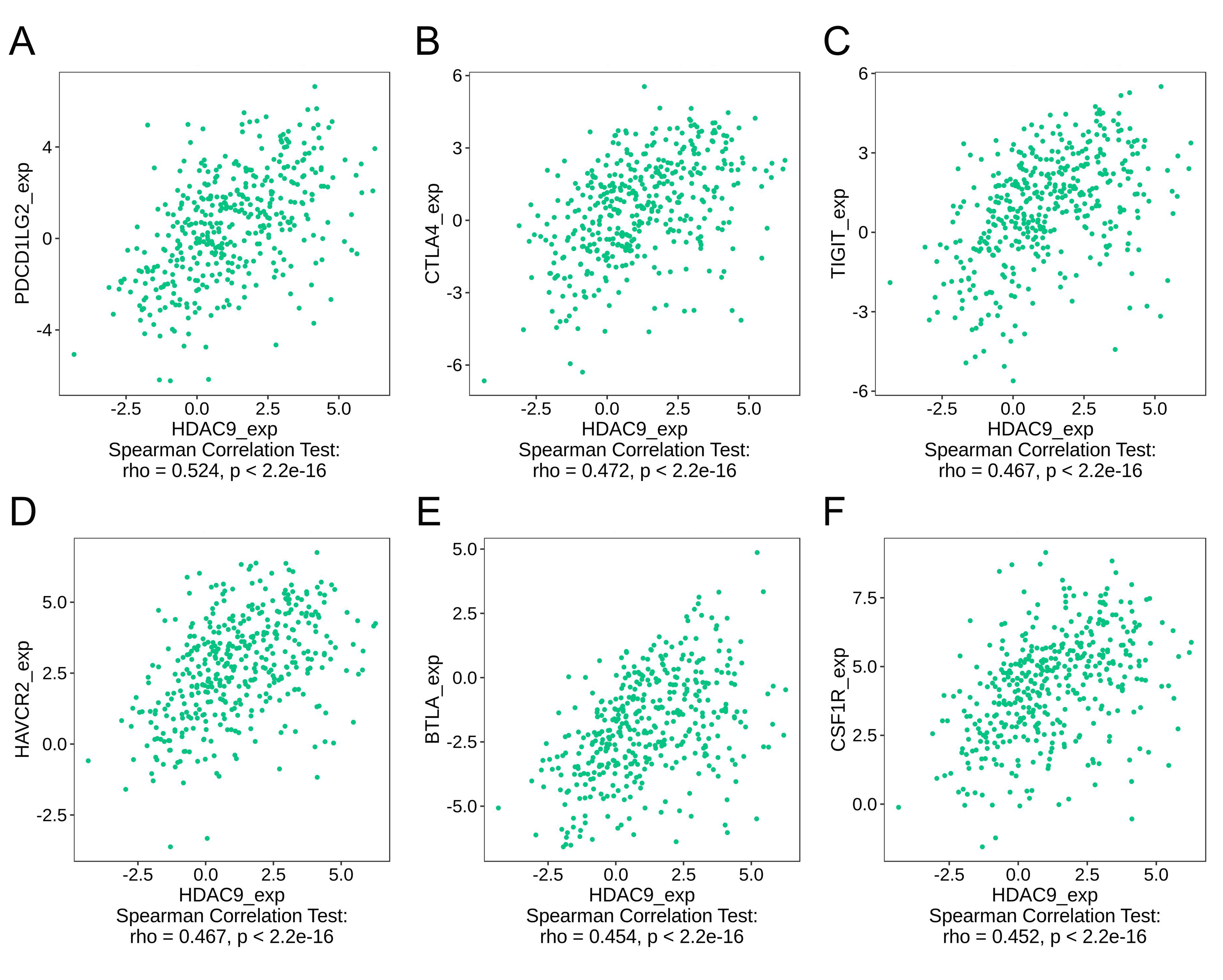

Supplement: S2 Fig — The first six immunosuppressive cytokines with the strongest correlation with HDAC9 in BC were visualized (A-F). HDAC9, histone deacetylase 9; BC, bladder cancer. (TIF) [file pone.0264527.s002.tif]

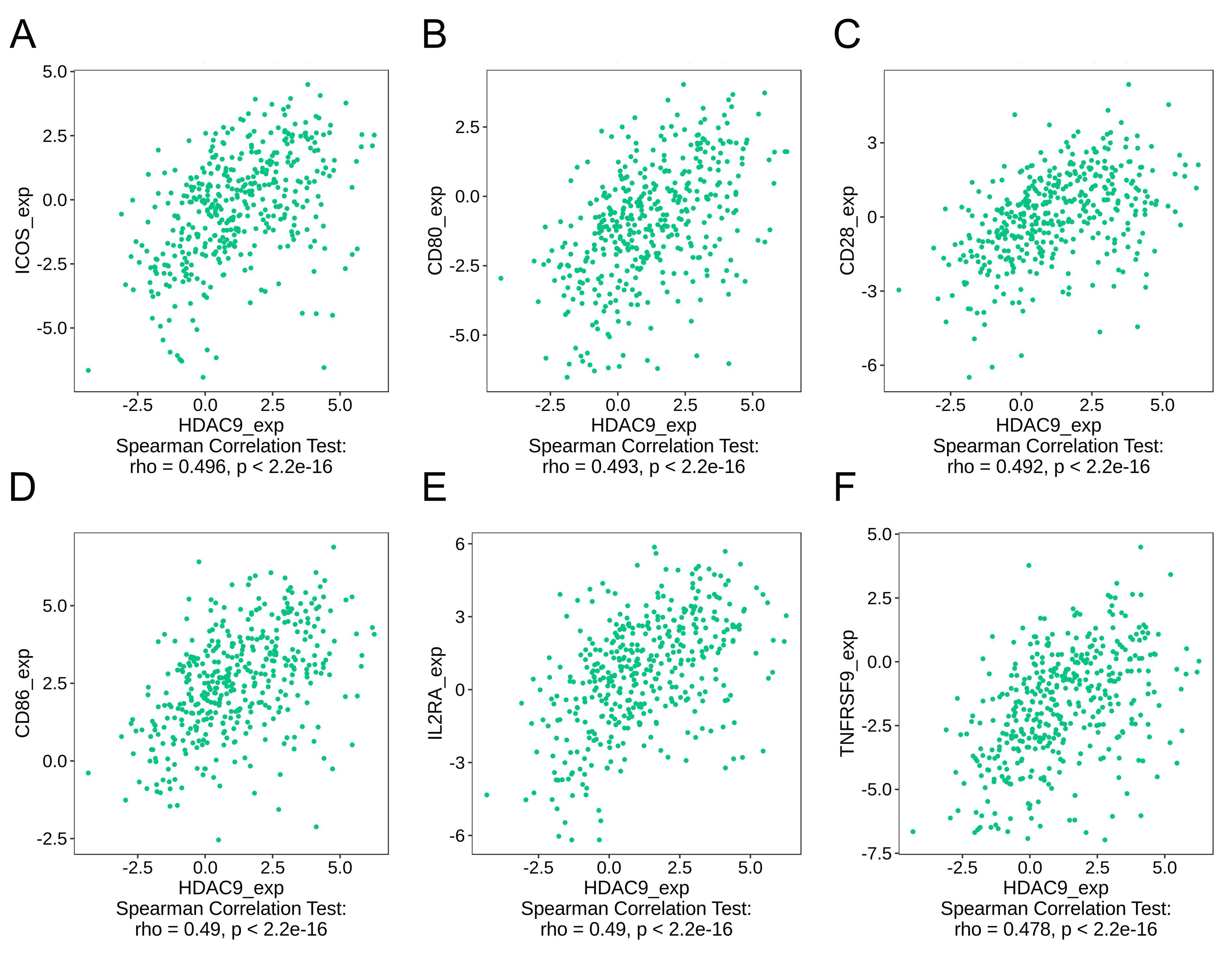

Supplement: S3 Fig — The first six immune-activating cytokines with the strongest correlation with HDAC9 in BC were visualized (A-F). HDAC9, histone deacetylase 9; BC, bladder cancer. (TIF) [file pone.0264527.s003.tif]

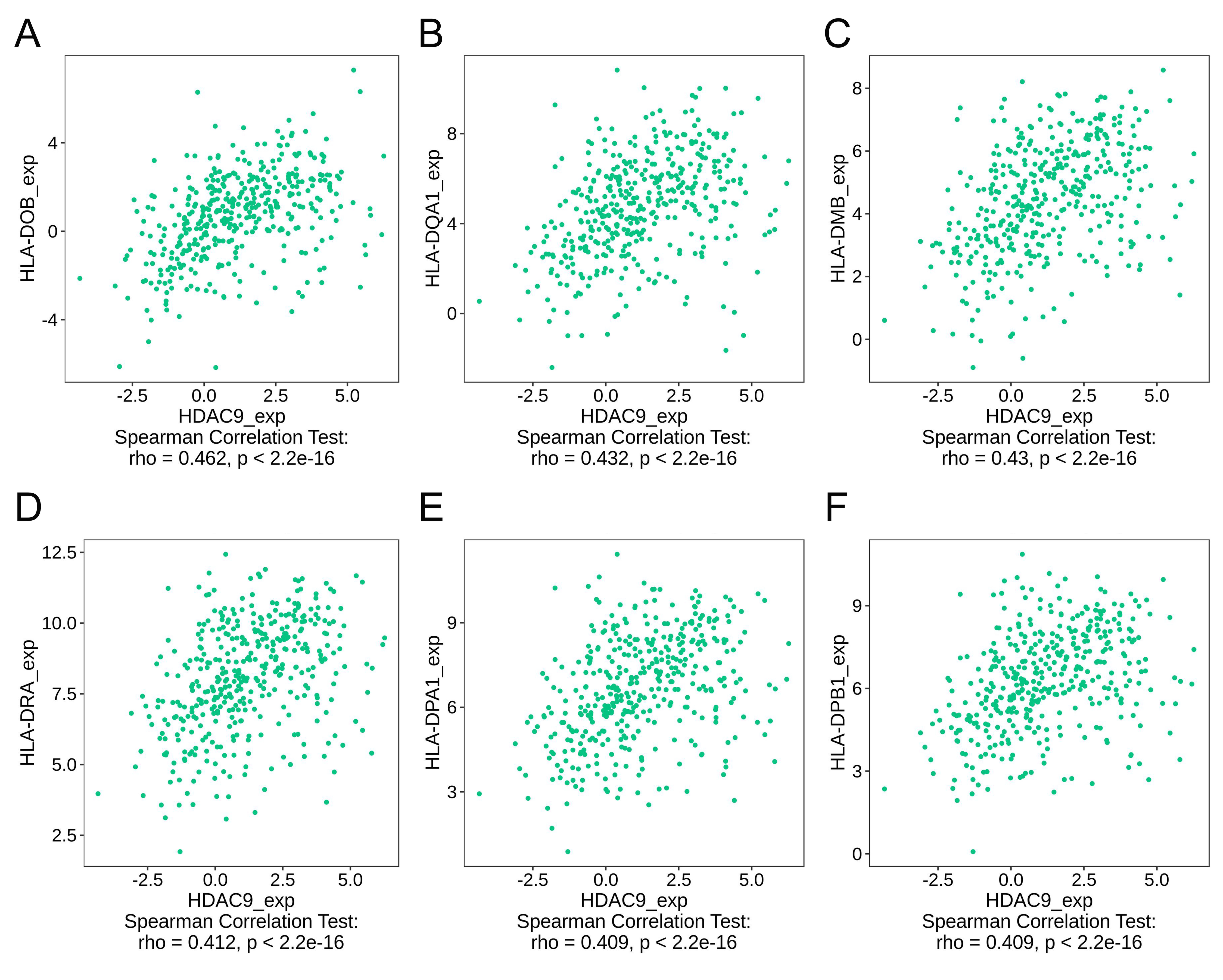

Supplement: S4 Fig — The first six immune-activating cytokines with the strongest correlation with HDAC9 in BC were visualized (A-F). HDAC9, histone deacetylase 9; MHC, major histocompatibility complex; BC, bladder cancer. (TIF) [file pone.0264527.s004.tif]

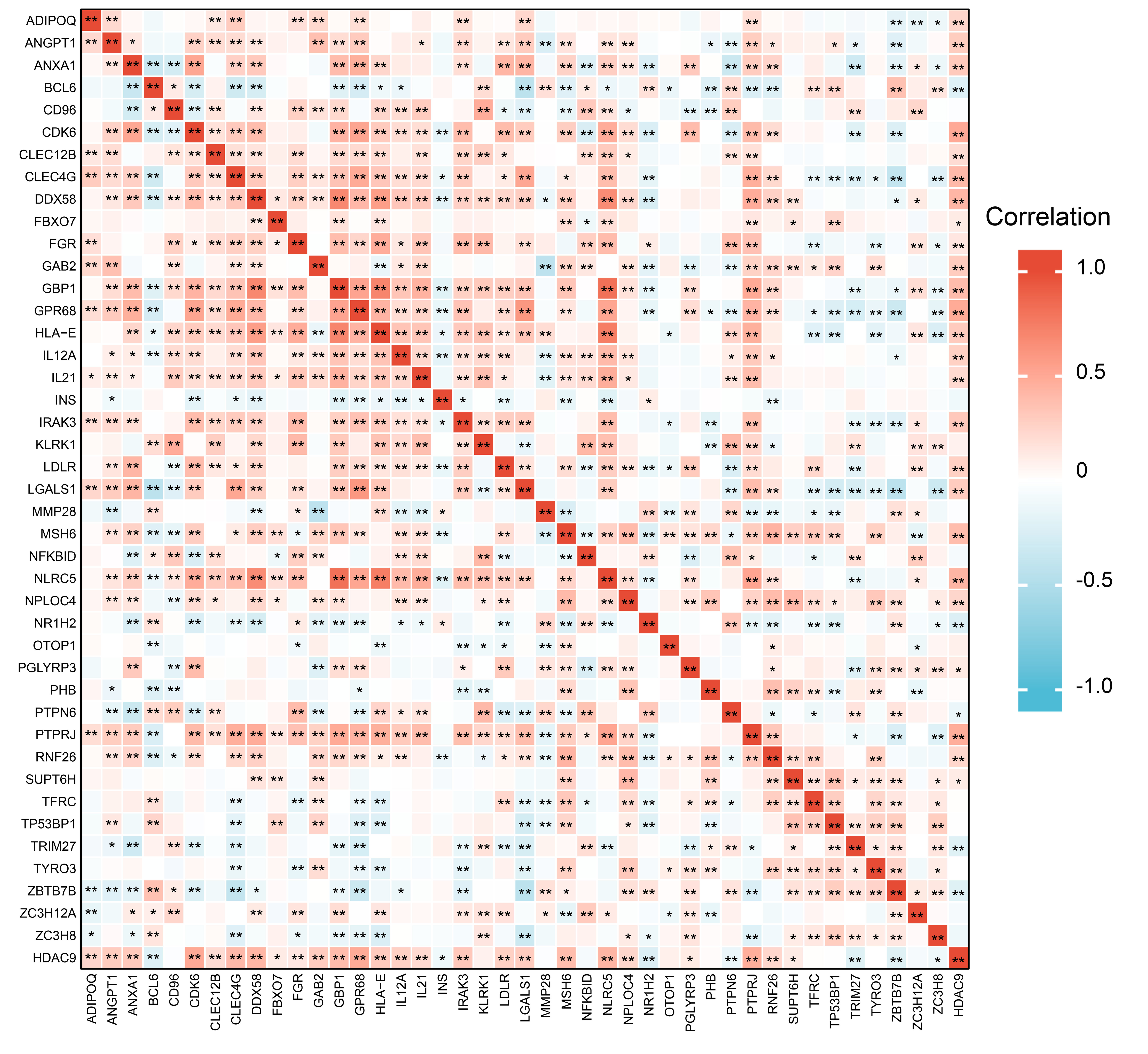

Supplement: S5 Fig — Coexpression analysis was performed to further understand connections between HDAC9 and the risk score genes. The relevant results were visualized via a heatmap. HDAC9, histone deacetylase 9; *, P. adjust <0.05; **, P. adjust < 0.01. (TIF) [file pone.0264527.s005.tif]

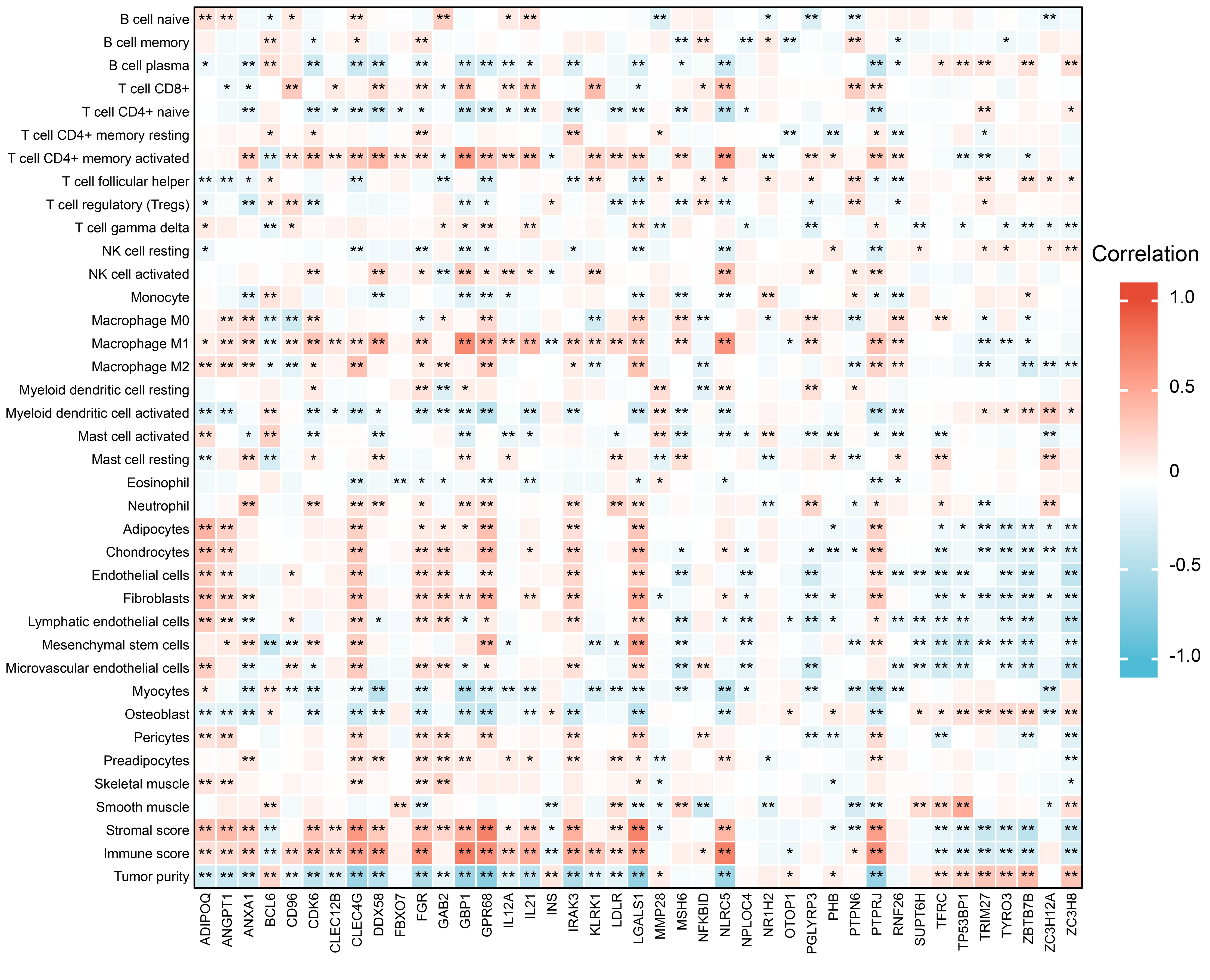

Supplement: S6 Fig — Correlation analysis was performed to further understand connections between genes included in the risk signature and TME. The relevant results were visualized via a heatmap. TME, tumor microenvironment; *, P. adjust <0.05; **, P adjust < 0.01. (TIF) [file pone.0264527.s006.tif]
