# Supplementary material for: An HDAC9-associated immune-related signature predicts bladder cancer prognosis
Source: PLoS One. 2022 Mar 3;17(3):e0264527. doi: 10.1371/journal.pone.0264527 (PMC8893690; doi:10.1371/journal.pone.0264527)
Supplement: S1 Table — (DOCX) [file pone.0264527.s007.docx]

**S1 Table** Genes related to survival were distinguished via univariable Cox regression.

| ID | HR | 95%CI | P.adj |
| --- | --- | --- | --- |
| ADIPOQ | 1.102 | 1.049 - 1.158 | 0.004 |
| ANGPT1 | 1.133 | 1.046 - 1.227 | 0.013 |
| ANXA1 | 1.002 | 1.001 - 1.003 | 0.006 |
| B2M | 1.000 | 0.999 - 1.000 | 0.020 |
| BATF | 0.982 | 0.970 - 0.994 | 0.016 |
| BCL3 | 0.987 | 0.976 - 0.999 | 0.043 |
| BCL6 | 0.967 | 0.944 - 0.992 | 0.020 |
| BST2 | 0.999 | 0.998 - 1.000 | 0.044 |
| BTN2A2 | 0.865 | 0.763 - 0.980 | 0.035 |
| CD274 | 0.954 | 0.912 - 0.997 | 0.043 |
| CD74 | 1.000 | 0.999 - 1.000 | 0.024 |
| CD96 | 0.841 | 0.768 - 0.921 | 0.004 |
| CDK6 | 1.062 | 1.018 - 1.109 | 0.019 |
| CLEC12B | 0.141 | 0.027 - 0.736 | 0.032 |
| CLEC4G | 1.061 | 1.026 - 1.098 | 0.006 |
| COL3A1 | 1.000 | 1.000 - 1.001 | 0.020 |
| CTLA4 | 0.866 | 0.777 - 0.965 | 0.020 |
| CXCL12 | 1.014 | 1.005 - 1.024 | 0.015 |
| DDX21 | 1.018 | 1.005 - 1.032 | 0.019 |
| DDX58 | 0.970 | 0.943 - 0.997 | 0.040 |
| DHX58 | 0.951 | 0.907 - 0.997 | 0.043 |
| DUSP10 | 1.009 | 1.000 - 1.018 | 0.046 |
| EXO1 | 1.063 | 1.004 - 1.125 | 0.043 |
| FBN1 | 1.046 | 1.018 - 1.074 | 0.008 |
| FBXO7 | 0.940 | 0.886 - 0.998 | 0.044 |
| FER | 1.445 | 1.087 - 1.920 | 0.023 |
| FGR | 0.887 | 0.813 - 0.969 | 0.019 |
| GAB2 | 1.075 | 1.023 - 1.131 | 0.017 |
| GATA3 | 0.997 | 0.994 - 0.999 | 0.027 |
| GBP1 | 0.988 | 0.980 - 0.996 | 0.015 |
| GPR68 | 1.025 | 1.001 - 1.049 | 0.043 |
| GPRC5B | 1.039 | 1.006 - 1.072 | 0.032 |
| GPS2 | 0.713 | 0.572 - 0.890 | 0.015 |
| HK1 | 1.013 | 1.001 - 1.026 | 0.043 |
| HLA-A | 0.999 | 0.999 - 1.000 | 0.019 |
| HLA-B | 1.000 | 0.999 - 1.000 | 0.019 |
| HLA-DMB | 0.974 | 0.951 - 0.997 | 0.038 |
| HLA-DRA | 1.000 | 0.999 - 1.000 | 0.029 |
| HLA-E | 0.998 | 0.997 - 0.999 | 0.008 |
| HLA-F | 0.989 | 0.982 - 0.997 | 0.016 |
| HTRA1 | 1.003 | 1.001 - 1.006 | 0.026 |
| IFNG | 0.781 | 0.630 - 0.969 | 0.035 |
| IL12A | 1.415 | 1.019 - 1.964 | 0.043 |
| IL12B | 0.060 | 0.004 - 0.942 | 0.046 |
| IL12RB1 | 0.872 | 0.767 - 0.992 | 0.043 |
| IL21 | 0.007 | 0.000 - 0.485 | 0.034 |
| IL31RA | 1.410 | 1.101 - 1.807 | 0.019 |
| INS | 1.083 | 1.034 - 1.133 | 0.006 |
| IRAK3 | 1.238 | 1.053 - 1.456 | 0.020 |
| IRF1 | 0.976 | 0.959 - 0.993 | 0.019 |
| KIR2DL4 | 0.615 | 0.433 - 0.875 | 0.019 |
| KITLG | 1.025 | 1.003 - 1.048 | 0.035 |
| KLRC1 | 0.390 | 0.197 - 0.773 | 0.019 |
| KLRK1 | 0.033 | 0.007 - 0.157 | 0.001 |
| LAG3 | 0.950 | 0.909 - 0.993 | 0.035 |
| LDLR | 1.024 | 1.008 - 1.040 | 0.016 |
| LGALS1 | 1.001 | 1.000 - 1.001 | 0.008 |
| LGALS9 | 0.978 | 0.964 - 0.993 | 0.015 |
| LRRC32 | 1.027 | 1.007 - 1.047 | 0.019 |
| MMP28 | 0.983 | 0.970 - 0.995 | 0.019 |
| MSH2 | 1.028 | 1.000 - 1.057 | 0.047 |
| MSH6 | 1.049 | 1.003 - 1.096 | 0.043 |
| NFKBID | 0.693 | 0.575 - 0.835 | 0.004 |
| NLRC5 | 0.914 | 0.860 - 0.972 | 0.016 |
| NMI | 0.964 | 0.933 - 0.997 | 0.041 |
| NOTCH2 | 1.037 | 1.009 - 1.065 | 0.020 |
| NPLOC4 | 1.038 | 1.006 - 1.071 | 0.032 |
| NR1H2 | 0.972 | 0.947 - 0.997 | 0.036 |
| OTOP1 | 1.245 | 1.015 - 1.528 | 0.043 |
| PARP14 | 0.977 | 0.956 - 0.999 | 0.043 |
| PARP3 | 0.959 | 0.924 - 0.995 | 0.035 |
| PDCD1 | 0.869 | 0.769 - 0.981 | 0.035 |
| PGC | 1.022 | 1.002 - 1.043 | 0.041 |
| PGLYRP3 | 1.031 | 1.012 - 1.051 | 0.011 |
| PHB | 1.023 | 1.008 - 1.039 | 0.015 |
| PPARG | 0.993 | 0.987 - 0.999 | 0.041 |
| PPP3CB | 1.061 | 1.007 - 1.118 | 0.036 |
| PRKDC | 1.027 | 1.010 - 1.044 | 0.011 |
| PRNP | 1.003 | 1.000 - 1.005 | 0.032 |
| PTK2B | 0.949 | 0.902 - 0.997 | 0.043 |
| PTPN6 | 0.926 | 0.896 - 0.957 | 0.001 |
| PTPRJ | 1.114 | 1.031 - 1.205 | 0.019 |
| PVR | 1.018 | 1.002 - 1.034 | 0.035 |
| RNF26 | 1.033 | 1.014 - 1.052 | 0.006 |
| SDC4 | 0.998 | 0.996 - 1.000 | 0.044 |
| SEC14L1 | 1.076 | 1.019 - 1.136 | 0.020 |
| SH2D1A | 0.836 | 0.700 - 0.997 | 0.046 |
| SLA2 | 0.828 | 0.707 - 0.971 | 0.032 |
| SLIT2 | 1.158 | 1.027 - 1.305 | 0.029 |
| ST3GAL1 | 0.978 | 0.958 - 0.999 | 0.046 |
| STXBP1 | 1.075 | 1.009 - 1.145 | 0.035 |
| STXBP2 | 0.954 | 0.923 - 0.986 | 0.019 |
| SUPT6H | 1.044 | 1.010 - 1.080 | 0.023 |
| TAPBPL | 0.972 | 0.946 - 0.999 | 0.044 |
| TBC1D10C | 0.904 | 0.822 - 0.994 | 0.043 |
| TBX21 | 0.583 | 0.393 - 0.864 | 0.019 |
| TFRC | 1.005 | 1.001 - 1.008 | 0.026 |
| TGFB3 | 1.030 | 1.001 - 1.060 | 0.044 |
| THBS1 | 1.004 | 1.001 - 1.008 | 0.025 |
| THY1 | 1.009 | 1.001 - 1.016 | 0.032 |
| TNFRSF14 | 0.941 | 0.910 - 0.972 | 0.005 |
| TP53BP1 | 1.131 | 1.014 - 1.262 | 0.036 |
| TRAFD1 | 0.970 | 0.947 - 0.994 | 0.027 |
| TRIM27 | 0.910 | 0.862 - 0.960 | 0.006 |
| TYRO3 | 1.138 | 1.056 - 1.226 | 0.006 |
| VTCN1 | 1.011 | 1.003 - 1.019 | 0.020 |
| ZBTB7B | 0.979 | 0.960 - 0.997 | 0.035 |
| ZC3H12A | 0.983 | 0.969 - 0.997 | 0.028 |
| ZC3H8 | 0.735 | 0.593 - 0.911 | 0.018 |
| ZFPM1 | 0.693 | 0.542 - 0.887 | 0.016 |

HR, hazard ratio; 95%CI, 95% confidence interval.
