# Supplementary material for: An HDAC9-associated immune-related signature predicts bladder cancer prognosis
Source: PLoS One. 2022 Mar 3;17(3):e0264527. doi: 10.1371/journal.pone.0264527 (PMC8893690; doi:10.1371/journal.pone.0264527)
Supplement: S2 Table — (DOCX) [file pone.0264527.s008.docx]

**S2 Table** The coefficients of included genes.

| ID | Coefficients |
| --- | --- |
| ADIPOQ | 0.04447 |
| ANGPT1 | 0.01257 |
| ANXA1 | 0.00087 |
| BCL6 | -0.00007 |
| CD96 | -0.00171 |
| CDK6 | 0.00287 |
| CLEC12B | -0.21707 |
| CLEC4G | 0.01956 |
| DDX58 | -0.01630 |
| FBXO7 | -0.01290 |
| FGR | -0.02504 |
| GAB2 | 0.00371 |
| GBP1 | -0.00607 |
| GPR68 | 0.01463 |
| HLA.E | -0.00072 |
| IL12A | 0.12151 |
| IL21 | -0.73505 |
| INS | 0.04197 |
| IRAK3 | 0.13857 |
| KLRK1 | -0.32989 |
| LDLR | 0.01007 |
| LGALS1 | 0.00013 |
| MMP28 | -0.00477 |
| MSH6 | 0.00084 |
| NFKBID | -0.10828 |
| NLRC5 | -0.01046 |
| NPLOC4 | 0.00933 |
| NR1H2 | -0.00394 |
| OTOP1 | 0.04879 |
| PGLYRP3 | 0.00445 |
| PHB | 0.00632 |
| PTPN6 | -0.00404 |
| PTPRJ | 0.06663 |
| RNF26 | 0.01166 |
| SUPT6H | 0.00989 |
| TFRC | 0.00105 |
| TP53BP1 | 0.01925 |
| TRIM27 | -0.03870 |
| TYRO3 | 0.03098 |
| ZBTB7B | -0.00064 |
| ZC3H12A | -0.00158 |
| ZC3H8 | -0.19248 |
